# WormBase 2016: expanding to enable helminth genomic research

**DOI:** 10.1093/nar/gkv1217

**Published:** 2015-11-17

**Authors:** Kevin L. Howe, Bruce J. Bolt, Scott Cain, Juancarlos Chan, Wen J. Chen, Paul Davis, James Done, Thomas Down, Sibyl Gao, Christian Grove, Todd W. Harris, Ranjana Kishore, Raymond Lee, Jane Lomax, Yuling Li, Hans-Michael Muller, Cecilia Nakamura, Paulo Nuin, Michael Paulini, Daniela Raciti, Gary Schindelman, Eleanor Stanley, Mary Ann Tuli, Kimberly Van Auken, Daniel Wang, Xiaodong Wang, Gary Williams, Adam Wright, Karen Yook, Matthew Berriman, Paul Kersey, Tim Schedl, Lincoln Stein, Paul W. Sternberg

**Affiliations:** 1 European Molecular Biology Laboratory, European Bioinformatics Institute, Wellcome Trust Genome Campus, Hinxton, Cambridge CB10 1SD, UK; 2 Informatics and Bio-computing Platform, Ontario Institute for Cancer Research, Toronto, ON M5G0A3, Canada; 3 Division of Biology and Biological Engineering 156–29, California Institute of Technology, Pasadena, CA 91125, USA; 4 Wellcome Trust Sanger Institute, Wellcome Trust Genome Campus, Hinxton, Cambridge CB10 1SA, UK; 5 Department of Genetics, Washington University School of Medicine, St. Louis, MO 63110, USA; 6 Howard Hughes Medical Institute, California Institute of Technology, Pasadena, CA 91125, USA

## Abstract

WormBase (www.wormbase.org) is a central repository for research data on the biology, genetics and genomics of *Caenorhabditis elegans* and other nematodes. The project has evolved from its original remit to collect and integrate all data for a single species, and now extends to numerous nematodes, ranging from evolutionary comparators of *C. elegans* to parasitic species that threaten plant, animal and human health. Research activity using *C. elegans* as a model system is as vibrant as ever, and we have created new tools for community curation in response to the ever-increasing volume and complexity of data. To better allow users to navigate their way through these data, we have made a number of improvements to our main website, including new tools for browsing genomic features and ontology annotations. Finally, we have developed a new portal for parasitic worm genomes. *WormBase ParaSite* (parasite.wormbase.org) contains all publicly available nematode and platyhelminth annotated genome sequences, and is designed specifically to support helminth genomic research.

## INTRODUCTION

Nematodes are the most abundant animals on the planet (1), and over 25 000 species have been described (2), displaying a remarkable diversity in terms of size, form, lifestyle, habitat and reproductive mode (3). *Caenorhabditis elegans* is a free-living nematode that has been used for over 50 years as a model system in experimental biology (4). Its transparency, reproductive mode, small size, rapid generation time, simple nervous system and invariant cell lineage have made it ideally suited to the study of animal genetics [(5); www.wormbook.org]. The mission of WormBase is to facilitate and accelerate biological research using *C. elegans* by making the collected outputs of the research community accessible from a single resource, and to enable the transfer of this wealth of knowledge to the study of other metazoa, from nematodes to humans.

The specific aims of WormBase are to: (i) place nematode data described in the research literature, deposited in the archives, or directly submitted to us into context via a combination of detailed manual curation and semi-automatic data integration; (ii) curate the reference genome sequence, gene structures and other genomic features for a small set of well-studied nematode species, thereby providing a high quality foundation for downstream studies and (iii) develop web displays and tools to allow users to easily visualize and query these data.

## CURATION STATUS

### Genomes and annotations for core species

WormBase acts as the guardian of the reference genome and annotation for *C. elegans* and a small number of additional ‘core’ species (www.wormbase.org/species/all), curating gene structures and other sequence features, assigning identifiers to primary annotation elements, and maintaining a complete historical record of all updates. In general, core status is reserved for species with a high-quality (chromosome-level) reference genome and identifiable research community that have expressed a desire for continued improvement of the genome sequence and annotation.

We previously reported on the adoption of the filarial parasitic nematode *Brugia malayi* as a WormBase core species, and described a concerted effort to annotate its genome to high quality using a combination of modern automated methods and manual curation (6). Since then, we have added two key parasitic nematodes to the core set: *Onchocerca volvulus*, a causative agent of onchocerciasis (river blindness), and *Strongyloides ratti*, the rat laboratory analogue of the causative agent of strongyloidiasis (threadworm infection). Both genomes have undergone first pass automatic gene structure annotation similar to that performed for the *B. malayi* genome, and are currently undergoing targeted manual curation of gene structures. We encourage users to report issues they find with the annotation of these genomes, and are committed to addressing them as a priority.

### Enabling community curation of the research literature

Comprehensive extraction and standardization of data from the *C. elegans* research literature remains one of the primary goals of the project. We target over 20 specific data types, and each presents its own challenge in terms of volume and ease of extraction. For some data types, such as gene expression patterns, curation keeps pace with the literature. For others, such as the phenotypes of mutant alleles and RNA-interference knockouts, the high volume of both historical publications and more recent high-throughput projects make the goal of keeping up-to-date unachievable with the resources we have. Help from the community is vital to address this shortfall, and we have recently worked on methods to allow researchers to contribute to curation for a small number of specific data types with the biggest backlog.

The three data-types that we have targeted are: (i) phenotypes of mutant alleles; (ii) molecular details for mutant alleles and (iii) textual gene summaries. Custom web-forms for each of these types have been designed to make the barrier to participation as low as possible (www.wormbase.org/about/userguide/submit_data). Authors of newly published papers are invited to submit their data via the forms, and researchers are also invited to review and update existing annotations. Submissions received via this system are prioritised for curator review and inclusion in WormBase.

The gene summaries referred to above are short paragraphs that provide an overview of the gene and its function, and include information about molecular identity, biological processes and pathways that the gene product participates in, temporal and spatial expression, and relationships with other genes via interaction or homology. Although WormBase users regard these summaries as highly valuable, the process of writing them is time-consuming. To address this, we have developed new software that automatically transforms structured data on gene function into natural language sentences, which are then collated to form a summary description. For example, the description created for the *C. elegans* gene *tbc-8* (www.wormbase.org/species/c_elegans/gene/WBGene00008018) is:

‘*tbc-8* is an ortholog of human SGSM2 (small G protein signaling modulator 2) and SGSM1 (small G protein signaling modulator 1); *tbc-8* is involved in dense core granule maturation; *tbc-8* exhibits Rab GTPase binding activity and is predicted to have GTPase activator activity, based on protein domain information; *tbc-8* is localized to the Golgi trans cisterna, the early endosome, the Golgi medial cisterna and the cytosol.’

The software has allowed us to create several thousand provisional gene summaries for *C. elegans* and other core species. As well as being useful in their own right, they also act as valuable and convenient starting points for manual revision.

## WORMBASE PARASITE

Beyond *C. elegans*, the nematode species covered by WormBase fall into one of two categories: free-living relatives of *C. elegans*; and plant and animal parasitic nematodes. For the former, the research interest is mainly in the area of comparative genomics and evolution of the ‘reference’ nematode *C. elegans*. The latter, however, have biomedical and agricultural importance and attract research interest in their own right. The primary research goal of parasitologists is to identify ways of controlling the parasite, and as such their desired entry points and common use-cases for WormBase are often distinct from those of scientists doing basic science using *C. elegans* as a model. Furthermore, the community of scientists working on parasitic worms (helminths) includes those working on platyhelminths (flatworms), which have historically been beyond the scope of WormBase. There have been recent concerted efforts to sequence the genomes of many helminths (e.g. the 50 Helminth Genomes initiative, www.sanger.ac.uk/science/collaboration/50HGP). Most of the genomes released so far are draft quality, and subject to high update frequency.

In response to these challenges, we have embarked on a systematic data integration effort for parasitic worms. The results can be viewed in *WormBase ParaSite* (parasite.wormbase.org), a new sub-portal of WormBase aiming to focus on the use-cases of scientists engaged in helminth genomics.

### Genomes and annotation

We have endeavoured to include all publicly available helminth genomes in WormBase ParaSite. Where multiple independent genome projects exists for the same species (e.g. for *Haemonchus contortus* (7,8)), and *Ascaris suum* (9,10)), we have included all. At time of writing we have genomes from 63 nematode species (71 genomes) and 27 platyhelminth species (28 genomes), and we maintain an up-to-date list of all genomes available through the site (parasite.wormbase.org/species.html).

These genome sequences have been collected from a variety of different sources, including the nucleotide archives, project-specific FTP sites, and direct engagement with genome project scientists. In all, 18 different groups were responsible for generating the data. In about half of the cases, we imported previously described or directly submitted gene structure annotations. For the remaining genomes, we used MAKER2 (11) to generate high-quality annotations by integrating evidence from multiple sources: *ab initio* gene predictions from AUGUSTUS (12), GeneMark-ES (13) and SNAP (14); projected annotations from *C. elegans* and the taxonomically nearest previously-annotated helminth using GenBlastG (15) and RATT (16); and alignments of ESTs, mRNAs and proteins from related organisms.

### Comparative genomics

We have used the Ensembl Compara system (17) to infer evolutionary histories for all helminth genes, supplemented by gene sets from 9 free-living species (including *C. elegans*) and 12 comparator species (including human and other model organisms). The result is the organisation of around 2.5 million genes into around 150 000 homology groups, each with a protein multiple alignment which is used to infer orthologous and paralogous relationships between the genes.

In addition to gene-based comparative analysis, we have also a produced a whole-genome multiple alignment for a subset of the collection, using Progressive Cactus (18,19). This alignment is made available in the form of a Hierarchical Alignment (HAL) file (20), and included in the WormBase UCSC Assembly Hub (see below). More genomes will be added to the alignment in the future.

### Electronic annotation of gene function

The research literature for most of the species in WormBase ParaSite is sparse, although we anticipate that the provision of the genomes will stimulate new research. In lieu of annotations based on experimental evidence, we have used established automated methods to predict the function of as many gene products as possible. First, to predict protein domains, assign terms from the Gene Ontology (GO), and classify the proteins into families, we have used InterProScan (21). Second, we have used the ortholog assignments from the Compara pipeline line to ‘project’ experimentally derived GO annotations and product names from well-annotated species (for example *C. elegans* and other model organisms).

### Infrastructure and tools

**Figure 1.**
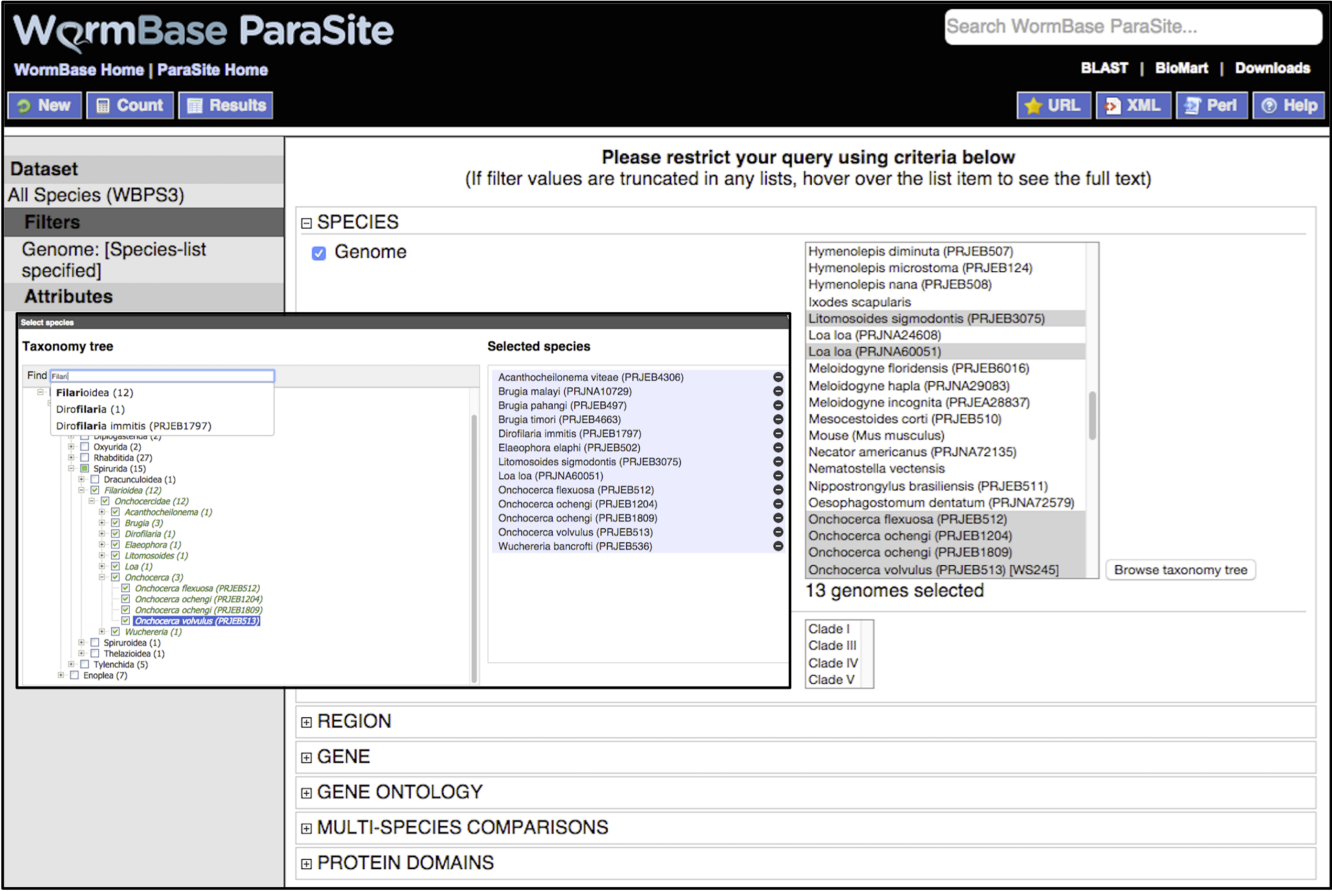
Querying multiple species in the WormBase ParaSite BioMart. A taxonomy-tree-based widget allows the selection of whole clades of species for querying, with an auto-complete feature allowing rapid identification of specific species or clades within the tree. The selection of species belonging to the Filarioidea nematode superfamily is shown.

The Ensembl infrastructure (22) is the basis for much of the management, analysis and display of data in WormBase ParaSite. This has allowed us to provide a number of tools developed for the Ensembl project with little or no modification, for example the Variant Effect Predictor (23), and REST application programming interface (24). Others we have customised for use in WormBase ParaSite. For example, we have modified Ensembl code to provide a sequence search service and BioMart data mining platform (25) that allow the interrogation of all species, or large sub-groups of species (e.g. all nematodes) at once in a single query (see Figure 1).

All data for WormBase ParaSite can be downloaded from our FTP site in a new folder (ftp://ftp.wormbase.org/pub/wormbase/parasite) that uses identical structure and naming conventions to the main FTP site (which now restricts to WormBase core species and genomic data sets from other free-living nematode species).

## WEBSITE AND TOOLS

The main website (www.wormbase.org) continues to focus on the central mission of WormBase, which is to serve scientists using *C. elegans* as a model system. Since we launched a new version of the site in 2012, we have continued refining the organization and presentation of data with the aim of making more information available at a single glance. Not only does this minimize the amount of mouse clicking required to access common data, it can also provide new insights not possible when examining data on a piecemeal basis.

### New genome browser

Browsing the genome is one of the most common tasks at WormBase, facilitating exploration of the genetic and physical maps, reagents, gene structures, candidate genes for forward genetic screens, and targets for reverse genetics. More recently, the genome browser has become the standard tool for the exploration of large-scale, genome-wide studies of gene expression and functional elements.

The current software driving the WormBase genome browser, GBrowse (26), was originally developed for the WormBase site and has now become a mainstay of many model organism database projects (27–31). Designed before the advent of large-scale genomic datasets and the types of queries users expect to levy against them, GBrowse has reached end-of-life development status. After conducting a due-diligence survey of available replacements, we have selected JBrowse (32) from the Generic Model Organism Database project (GMOD, www.gmod.org) to replace GBrowse in WormBase. JBrowse represents a significant advancement over GBrowse, being much faster when browsing large regions, large datasets, or many tracks at concurrently. It also shares many of the user interface elements with GBrowse, providing a shallow learning curve for WormBase users.

JBrowse provides a number of features not present in GBrowse. For example, it allows users to view their own data in the browser without requiring the files to be uploaded to the server. Instead, the browser can be pointed to local file or a URL specifying the location of a remote file, and JBrowse will render the data by processing it locally. This is made possible due to the ‘in browser’ execution of JBrowse; all of the software to process the data and display it is included in the JavaScript that is downloaded when the user first accesses the tool. Another new feature allows users to perform a degree of in-browser data analysis by combining data in tracks using arithmetic and set operations, for example finding the union, intersection or exclusive or (XOR) of two tracks. Combination tracks can be used as input to other combination tracks, allowing users to build up arbitrarily complex analysis tracks.

We have created JBrowse browsers for all core species in WormBase, and these are currently available alongside GBrowse (accessible from the Tools menu, and also as an alternative view on the Location panel on each Gene page). This allows users to migrate to the new tool at their own pace.

### New ontology browser

WormBase annotates genes with terms from established ontologies for anatomy (33) (for spatial expression), disease (34) (for human disease relevance), life stage (for temporal expression) and phenotype (35) (for perturbation outcome), as well as gene function using the Gene Ontology (36). We have added a graphical tool to make it easier for users to navigate between related terms in these ontologies, and to quickly retrieve genes annotated with specific terms. The WormBase Ontology Browser (WOBr) is adapted from AmiGO 2, which uses Apache Solr to store and index the Gene Ontology and annotations made with it (36). We have generalised the build procedure to allow the loading of other ontologies and annotations, and created an index for each ontology used by WormBase.

**Figure 2.**
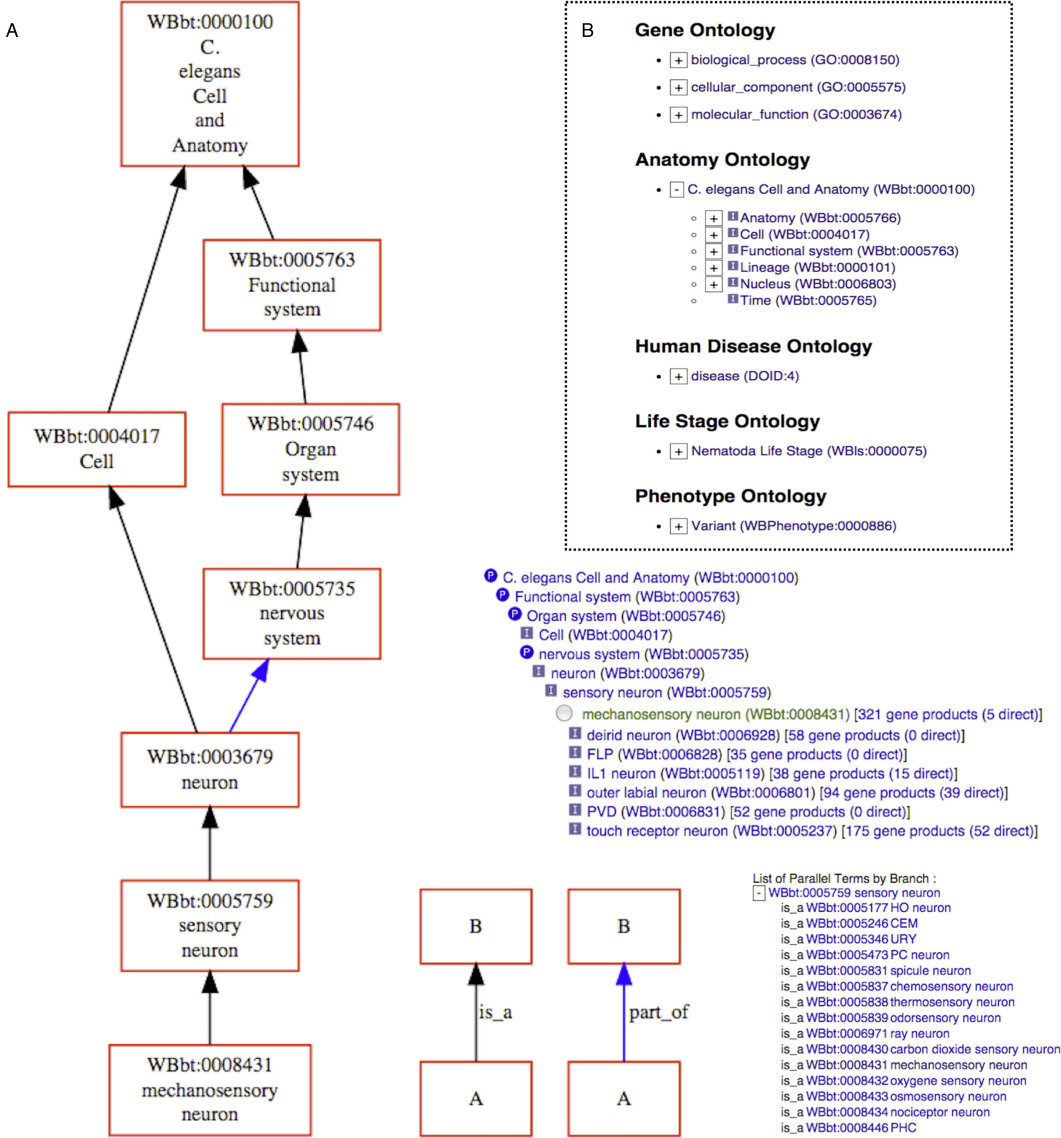
The WormBase Ontology Browser. (**A)** view of the anatomy term ‘mechanosensory neuron’. The sub-graph of the term and its ancestors in the hierarchy are depicted graphically to the left. To the right, the inference tree view of the term is shown, with icons indicating relationships in reference to the focus term (P = Part-of, I = Is-a). Numbers to the right are counts of genes annotated with that term (either directly or by inheritance). A list of parallel (sibling) terms to the focus term can also be viewed (bottom right). (**B**) main entry page for the ontology browser, which allows top-down navigation from the root term for each WormBase ontology.

Building on these indexes, we have developed two interfaces for exploring ontology annotations: (i) a view for each ontology term, which shows the relevant sub-graph of related terms as an interactive tree and (ii) an interactive browser, which allows root-to-leaf navigation of the complete directed acyclic graph for an ontology (see Figure 2).

### Data mining platforms

The primary data mining platform in WormBase is WormMine, our custom deployment of the InterMine data warehousing software (37). We have recently migrated WormMine to the Amazon Elastic Compute Cloud (AWS EC2), facilitating more frequent updates and greater performance. All WormBase core species are queryable in WormMine. In forthcoming releases we are committed to deepening available data, first to RNA interference experiments and generic access to genomic sequence, and later to all data classes available through WormBase.

We also provide a separate data mining platform for the WormBase ParaSite portal, using the BioMart technology (38). Whereas WormMine aims to provide access to a rich set of data for a small number of core species, ParaSite BioMart aims to provide simple access to a small set of basic data types (e.g. sequence, orthologs, cross references) for all nematode and platyhelminth genomes, and has been optimised to allow the querying of many species at once (Figure 1).

## OUTREACH

WormBase fosters a close relationship with its primary users, the *C. elegans* research community, collecting information on every active nematode laboratory and researcher, and connecting data and research outputs to the scientists that reported them. We continue to implement modern and engaging ways to communicate with our users. Our community forum (forums.wormbase.org) for discussion on general issues of nematode biology now has nearly 2000 members. We also continue to draw attention to interesting events and data sets in WormBase with our blog (blog.wormbase.org) and Twitter feed (www.twitter.com/wormbase).

To expand outreach efforts and engage more closely with our users, we have recently added a new in-line real-time chat feature to our website. Once initiated, users are connected directly with a WormBase staff member. Chat transcripts are automatically posted to our help desk issue tracker to verify that questions and comments are addressed satisfactorily. If a chat attempt is made whilst no operator is online, questions are posted directly to our help desk queue (as always, it is WormBase policy to acknowledge all queries within a 24 h window). Whilst chatting, users can freely navigate to other areas of the website, and even share their screen with WormBase staff to discuss issues. This feature provides us with a rapid way to address common questions or concerns so that users can continue their research without waiting for a help desk response via email.

## INTEGRATION WITH OTHER RESOURCES

An important remit of WormBase is the dissemination of project outputs beyond our own websites. We submit sequence annotations to the International Nucleotide Sequence Database Collaboration (INSDC) (39), and have a formal partnership with the Ensembl (22) and Ensembl Genomes (40) projects, working to ensure that up-to-date data for nematode genomes is displayed in those resources.

To further increase the range of resources in which WormBase data can be browsed, we have implemented a WormBase Assembly Hub. Assembly Hubs (41) are an emerging standard for the representation and remote hosting of data that can be displayed by genome browsers. The WormBase Assembly Hub (www.wormbase.org/about/userguide/wormbasehubs) is updated with every WormBase release, and allows, for the first time, current WormBase genomes and annotations to be viewed in the UCSC genome browser (41).

## FUTURE PLANS

As we reported previously (6), we are embarked on a major re-design of the back-end infrastructure and curation tools for WormBase. This is a long-term project, but we plan to roll out elements of this in the coming year in a way that will cause no disruption to users.

Increasing the utility of WormBase for biomedical research continues to be a priority for the project. We annotate *C. elegans* genes that have relevance to the study of human disease (42), curating descriptions of the association and forming connections to terms from the Disease Ontology (34) and to disease and gene records in the Online Mendelian Inheritance in Man (OMIM) (43). We plan to take this further by associating mutant alleles in *C. elegans* with orthologous genomic variants in human, using a combination of programmatic data integration and manual curation. We envision that this will be particularly useful for the study of human variants of unknown significance. On the specific issue of human disease caused by parasitic worms, we plan to augment WormBase ParaSite with additional features for the identification of putative targets for anti-helminthic drugs, for example by linking gene products to the ChEMBL (44) database of medicinal chemistry.
